# Dapagliflozin-affected endothelial dysfunction and altered gut microbiota in mice with heart failure

**DOI:** 10.7717/peerj.15589

**Published:** 2023-07-26

**Authors:** Nandi Bao, Xiaoli Liu, Xiaoling Zhong, Shuangshuang Jia, Ning Hua, Li Zhang, Guoxin Mo

**Affiliations:** 1 Chinese PLA General Hospital, Beijing, China; 2 First Medical Center of Chinese People’s Liberation Army (PLA) General Hospital, Beijing, China; 3 South China University of Technology, Guangzhou, China; 4 Anhui Medical University, Hefei, China; 5 The Eighth Medical Center of PLA General Hospital, Beijing, China

**Keywords:** Heart failure, Gut microbiota, Dapagliflozin, 16S rDNA sequencing, SGLT-2 inhibitors

## Abstract

**Aim:**

To investigate the potential microbiome profile of a mouse model with heart failure (HF) during dapagliflozin treatment.

**Method:**

An HF model was constructed in 8-week-old male mice, and cardiac tissues were analyzed using histological staining. Hemodynamic indexes were measured, and fecal samples were collected for 16S rDNA sequencing. Chao1, Shannon, and Simpson were used for α-diversity analysis. b-Diversity analysis was conducted using principal coordinate analysis (PCoA) and non-metric multidimensional scaling (NMDS) based on the Bray–Curtis distance. Linear discriminant analysis coupled with effect size measurements (LEfSe) was used to identify signature gut microbiota, and phylogenetic investigation of communities by reconstruction of unobserved states (PICRUSt) was used to predict the function of altered gut microbiota.

**Result:**

Dapagliflozin treatment reduced inflammation, infarction area, and cardiac fibrosis in HF mice. It also increased endothelial-dependent dilation and inflammation in mice with HF. Dapagliflozin decreased the ratio of Firmicutes/Bacteroidetes, which was increased in HF mice. There was no significant statistical difference in α-diversity among the control, HF, and HF+dapagliflozin groups. *Desulfovibrio*, *AF12*, and *Paraprevotella* were enriched in HF+dapagliflozin, while *Rikenella* and *Mucispirillum* were enriched in HF based on LEfSe. KEGG analysis revealed that altered gut microbiota was associated with fermentation, amino acid biosynthesis, nucleoside and nucleotide biosynthesis/degradation, fatty acid and lipid biosynthesis, carbohydrate biosynthesis/degradation, and cofactor/prosthetic group/electron carrier/vitamin biosynthesis.

**Conclusion:**

Understanding the microbiome profile helps elucidate the mechanism of dapagliflozin for HF. The signature genera identified in this study could be used as a convenient method to distinguish between HF patients and healthy individuals.

## Introduction

According to the latest definition from the European Society of Cardiology, heart failure (HF) is a complex clinical syndrome characterized by cardinal symptoms such as dyspnea, peripheral edema, increased jugular venous pressure, and fatigue. These symptoms are attributed to abnormalities in the structural and/or function of the myocardium (McDonagh et al., 2021). HF is the major cause of hospitalization and mortality in older adults. The Heart Failure Association Atlas reports (Seferovic et al., 2021) that the median incidence and median prevalence of HF are 3.2/1,000 person-years and 17.20/1,000, respectively. Age is the most significant risk factor for HF, with the incidence and prevalence of HF increasing with age. The American Heart Association concluded (Mozaffarian et al., 2016) that the proportion of older men (age ≥ 60) with HF is 6.6–10.6%, while for men (age 20–59), it is 0.2–1.5%. A similar trend is observed in adult women (proportion of age ≥60 women with HF: 4.8–13.5%; the proportion of age 20–59 women with HF: 0.6–6.6%).

HF is not due to a single pathogenic mechanism. A series of hemodynamic events lead to HF, such as dysfunction in heart rate, cardiac output, and filling pressure. Interestingly, there is a newly-discovered mechanism of the gut-to-heart axis based on the microbiome. The HF-induced ischemia and insufficient blood flow to internal organs are responsible for the impaired intestinal barrier, which results in the translocation and alteration of gut microbiota. This alteration causes impaired metabolism and inflammation in patients with HF (Zhang et al., 2021). Cui et al. (2018) found that patients with chronic heart failure (CHF) showed sharp alterations in gut microbiota composition. They concluded that *Ruminococcus gnavus* was enriched in CHF while *Faecalibacterium prausnitzii* was reduced; gut microbiota alteration resulted in an imbalance between protective metabolites and harmful metabolites. Hayashi et al. (2021) suggested the reduction of essential amino acids and histidine in HF might be related to the abnormal abundance of gut microbiota that contributes to the degradation and biosynthesis of these substances. Li et al. (2021) reported that HF is accompanied by a marked increase in Firmicutes/Bacteroidetes and identified 17 signature microbiota that was capable of identifying HF. Importantly, they suggested that alterations in genera were involved in the production of short-chain fatty acid and trimethylamine N-oxide. Microbiota alterations potentially encode and modulate metabolite production under the intestinal barrier, thereby evoking inflammation and immune response during the progression of HF. Understanding the profile of gut microbiota in HF will provide novel insight into the pathogenesis of this disease.

Dapagliflozin is a sodium-glucose cotransporter-2 (SGLT2) inhibitor known for its high efficiency, reversibility, and selectivity in glycemic control and blood pressure (Dhillon, 2019). Animal studies have shown that exposure to dapagliflozin alters the abnormality of global longitudinal systolic strain (GLS) in an animal model of HF (Withaar et al., 2021). Additionally, dapagliflozin has demonstrated its protective role in patients with HF. A phase-3 clinical trial (McMurray et al., 2019) showed dapagliflozin repressed the risk of the first worsening HF event compared with placebo treatment (10% *vs*. 13.7%; hazard ratio, 0.70; 95% CI [0.59–0.83]). Therefore, dapagliflozin treatment is instrumental in patients with HF. However, dapagliflozin’s effect on gut microbiota in cardiovascular disease is also noteworthy. Lee et al. (2018) observed marked alterations in the abundances of *Actinobacteria*, *Bacteroidetes*, *Firmicutes*, *Proteobacteria*, and *Verrucomicrobia* in response to dapagliflozin treatment in type 2 diabetic mouse model. Furthermore, a study in rat models with cardiovascular disease (Yang et al., 2020) showed that *Lachnoapiraceae*, *Desulfovibrionaceae*, *Oscillibacter*, and *Ruminiclostridium_9* might be potential therapeutic targets of dapagliflozin. Oh et al. (2019) concluded that dapagliflozin treatment can reduce the F/B ratio, significantly decrease *Adlercreutzia* and *Alistipes*, and increase *Streptococcus*. Thus, dapagliflozin treatment may improve intestinal disorder in patients with HF by targeting microbiota-associated metabolites in addition to modulating SGLT2. The relationship between dapagliflozin, gut microbiota, and heart function requires further investigation.

Therefore, we investigated whether dapagliflozin could treat HF by targeting gut microbiota using a mouse model of heart failure. We collected fecal samples from dapagliflozin-treated C57BL/6N mice to determine the potential microbiome profile that could help identify the indistinguishable symptoms of HF.

## Materials and Methods

### Animal experiment

Male 8-week-old C57BL/6N mice (Vitalriver, Beijing, China) weighing 18–20 g were housed on a 12 h light/dark cycle and given *ad libitum* access to food and water. Animal experiments were approved by the Animal Care and Use Ethic Committee (Approval No.: MDKN-2022–024). Following a 5-day-acclimatization, mice were randomly assigned to one of the three groups: control (*n* = 10), HF (*n* = 10), or HF+dapagliflozin (*n* = 10). The control group underwent thoracotomy without the ligation of the left anterior descending coronary artery (LAD) after being anesthetized with 100% oxygen and 1–4% isoflurane at 0.5 L/min. The HF group underwent thoracotomy with permanent ligation of 6–0 nonabsorbable silk sutures for 8 weeks after anesthesia (Maruyama et al., 2021). Mice in control and HF groups underwent i.p. injection of normal saline. The HF+dapagliflozin group received intraperitoneal injections of dapagliflozin (1 mg/kg/day) until the end of the 8 weeks after LAD ligation. The sample size in each group was determined using PASS software based on pre-experiment. Heart failure was confirmed in mice using an electrocardiogram monitor based on ST elevation/reduction before and after ligation. At the end of the 8-week period, mice were euthanized using intraperitoneal injection of 150 mg/kg pentobarbital sodium, and exsanguination was performed using cardiac puncture to collect serum samples. Left ventricular (LV) mass and body mass were measured, and heart samples were imaged using a camera (Nikon, Tokyo, Japan). Mice were euthanized by intraperitoneal injection of 150 mg/kg pentobarbital sodium at the end of the experiments or when reaching human endpoints if they showed signs of weight loss, appetite loss, depression, anxiety, infection, organ failure or if they became moribund. Mice were included in the HF and HF+ Dapagliflozin groups if they underwent successful LDA ligation, defined by a decreased cardiac motion amplitude such as heart rate, LVEF, and FS compared to healthy mice. Similarly, surviving mice were included in the control group after the sham operation. Mice were excluded if they died prematurely. Mice were grouped using the table of random numbers. In each group, five mice were used for tests such as Doppler ultrasound, endothelial-dependent dilation, ELISA, *etc*., while five mice were used for 16S rDNA analysis. The cross-control experiment was used in the present study. Animals and observers were blinded to medication treatment assignment; independent evaluators for RNA-sequencing were also blinded to experiment design.

### Doppler ultrasound

Cardiac function was measured weekly using Doppler ultrasound. Mice received 100% anesthesia oxygen and 1–4% isoflurane at 0.5 L/min for 15 min before the measurement. Left ventricular end-diastolic volume (LVEDV), left ventricular end-systolic volume (LVESV), fractional shortening (FS), and left ventricular ejection fraction (LVEF) in mice were measured.

### Endothelial-dependent dilation

We investigated the changes in endothelial function using the vascular tension method based on acetylcholine (Ach). Mice were euthanized by intraperitoneal injection of 150 mg/kg pentobarbital sodium. As previously described (Battson et al., 2017), secondary branches of the coronary artery were then fixed in pressure myograph chambers (DMT, USA) filled with warm physiologic saline solution and maintained at 37 °C under a pressure of 50-mmHg pressure for 1 h. The constricted arteries were then incubated with 10^−9^ to 10^–5^ M acetylcholine following the induction of phenylephrine at concentrations ranging from 10^−9^ to 10^−5^ M.

### Enzyme-linked immunosorbent assay

Serum sampling was performed on mice after they received an intraperitoneal injection of 150 mg/kg pentobarbital sodium. Serum samples were intraperitoneally collected from mice after 12-h fasting and analyzed using a microplate reader (Thermo Fisher, Waltham, MA, USA) at 450 nm based on enzyme-linked immunosorbent assay (ELISA) to obtain the concentration of cytokines, including MCP-1 (ab208979; Abcam, Waltham, MA, USA), IL-1β (ab197742; Abcam, Waltham, MA, USA), IL-6 (ab222503; Abcam, Waltham, MA, USA), and IL-17 (ab199081; Abcam, Waltham, MA, USA).

### Histological staining

Heart samples were collected from mice after they were subjected to an intraperitoneal injection of 150 mg/kg pentobarbital sodium. To determine the area of myocardial infarction (MI), three heart samples from each group were frozen at −20 °C for 30 min and cut into 3-mm-thickness sections. These sections were then stained using 2% 2,3,5-Triphenyltetrazolium chloride (TTC) (Solarbio, Beijing, China) for 15 min at 37 °C in the dark, and fixed with 4% paraformaldehyde (Thermo Fisher, Waltham, MA, USA) for 2 h. The area of MI was analyzed by Image J. Additionally, histopathology sections (thickness: 3 mm) of one frozen sample from each group were fixed in 4% paraformaldehyde for 10 min and then immersed in distilled water for 2 min. The sections were then stained with hematoxylin (H) for 10 min and eosin (E) staining for 2 min. Sections stained by H&E (Solarbio, Beijing, China) were sealed using neutral balsam after dehydration and transparency. Histopathological changes were observed under an optical microscope (Olympus, Tokyo, Japan). Collagen deposition in myocardial tissues was determined using Masson’s Trichrome Stain Kit (Solarbio, Beijing, China).

### Western blotting

Myocardial tissues were collected from mice after they were subjected to an intraperitoneal injection of 150 mg/kg pentobarbital sodium. The myocardial tissue was homogenized by T-PER tissue Protein Extraction Reagent (Thermo Fisher, Waltham, MA, USA) for 10 min, followed by centrifugation at 10,000×*g* for 5 min. The total protein in the supernatant was measured by a BCA protein assay kit (Pierce, Appleton, WI, USA) to determine protein concentration. Forty micrograms of total protein were separated by SDS-PAGE using an electrophoresis system (Bio-Rad, Hercules, CA, USA) and then transferred from the gel to PVDF membranes by a blotting system in an ice bath (Bio-Rad, Hercules, CA, USA). The membranes were incubated with the blocker BSA in TBS (Thermo Fisher, Waltham, MA, USA) for 2 h at room temperature, followed by overnight incubation with primary antibodies at 4 °C. Secondary antibody (ab97051, 1:10,000; Abcam, Waltham, MA, USA) was then used to culture the membranes for 1 h at 37 °C. Finally, membranes were incubated with SuperSignal West Pico Chemiluminescent Substrate (Thermo Fisher, Waltham, MA, USA) for 5 min before the X-ray film (Bio-Rad, Hercules, CA, USA) image was used to image the blots. Glyceraldehyde-3-phosphate dehydrogenase (GAPDH) was used as an internal control for proteins. The primary antibodies used included α-smooth muscle actin (α-SMA; ab5694, 2 μg/mL), fibronectin (FN; ab268020, 1/1,000), collagen I (ab270993, 1/1,000), collagen III (1/1,000, ab184993), connective tissue growth factor (CTGF; ab209780, 1/1,000), and GAPDH (ab8245, 1/10,000) that were purchased from Abcam (Waltham, MA, USA).

### Fecal collection and 16S rDNA sequencing and microbiome bioinformatics

We collected f samples of five mice in each group in 8^th^ week and extracted microbiota DNA from these samples using a microbiome DNA Purification kit (PureLink, Fair Lawn, NJ, USA). Paired-end 16S rDNA sequencing was performed on an Illumine NovaSeq platform. The forward primer for the V3–V4 region of the 16S rDNA was 5′-ACTCCTACGGGAGGC AGCA-3′ and the reverse primer of the V3–V4 region was 5′-GGACTACHVGGGTWTCTAAT-3′. Microbiome bioinformatics was performed with QIIME 2 2019.4 (https://docs.qiime2.org/2019.4/tutorials/). Raw sequences were demultiplexed using the Demux plugin, followed by primer trimming using the cutadapt plugin. The resulting sequences were merged, filtered, and dereplicated using Vsearch (https://github.com/torognes/vsearch/wiki/VSEARCH-pipeline). Taxonomy was annotated according to the Greengenes database (Release 13.8, http://greengenes.secondgenome.com/). The rarefaction of OTUs was performed by qiime feature-table rarefy. Based on QIIME2 and ggplot2 (https://ggplot2.tidyverse.org/), α-diversity of gut microbiota was calculated using Chao1 (increased Chao1 indicated the higher richness of gut microbiota), Simpson (increased Simpson suggested the more abundant diversity of microbiota), and Shannon (increased Shannon suggested the more abundant diversity of microbiota). Bray-Curtis method was used to calculate the sample distance and principal coordinate analysis (PCoA) (analyzed by ape, http://ape-package.ird.fr/) and non-metric multidimensional scaling (NMDS) (analyzed by Vegan, https://cran.r-project.org/web/packages/vegan/) were performed for β-diversity. The biomarkers of each group were determined using linear discriminant analysis coupled with effect size measurements (LEfSe) (performed by Python LEfSe, https://pypi.org/project/lefse/). The function of altered gut microbiota was investigated using the Phylogenetic Investigation of Communities through Reconstruction of Unobserved States (PICRUSt) tool. The enrichment analysis of metabolism pathways was performed based on KEGG, MetaCyc, and COG database using metagenomeSeq. Hierarchical clustering was performed according to the unweighted pair-group method with arithmetic means using the ggtree tool (https://bioconductor.org/packages/release/bioc/htmL/ggtree.html).

### Data analysis

We used SPSS20.0 (IBM, Armonk, NY, USA) to calculate the mean and SEM for the experimental data. We performed the Kruskal–Wallis test to analyze the statistical differences among multiple groups. A *p*-value < 0.05 at a 95% confidence interval was considered statistically significant. The *p*-value in LEfSe was adjusted using the Benjamini & Hochberg methods (Benjamini & Hochberg, 2018). All experiments were performed in triplicates.

## Results

### Dapagliflozin treatment affected endothelial dysfunction in HF

**Figure 1:**
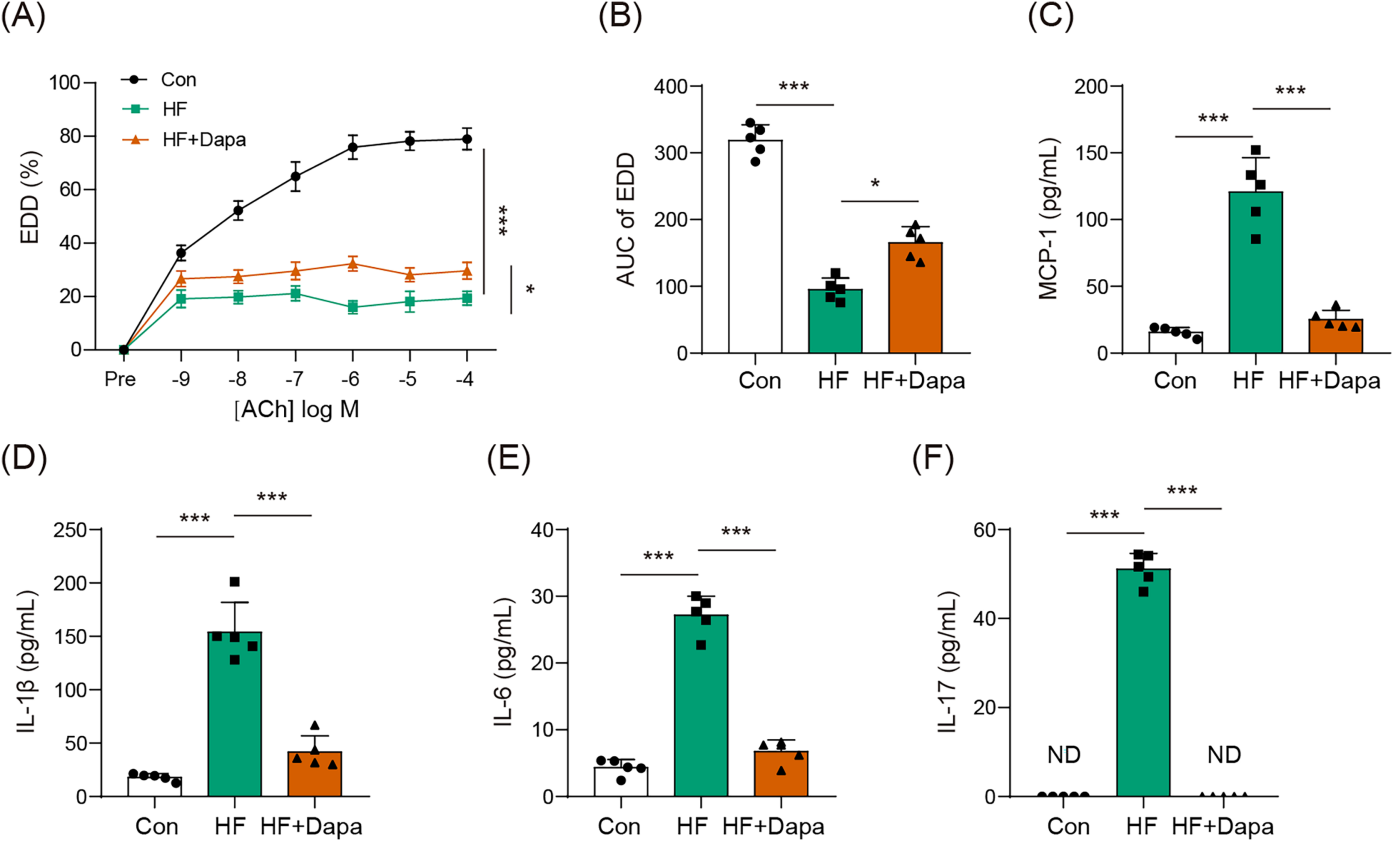
Dapagliflozin affected endothelial dysfunction in HF (*n* = 6). *p < 0.05 and ***p < 0.001.

Endothelial dysfunction is a prevalent cardiovascular event in HF. We explored the role of dapagliflozin in improving endothelial dysfunction in animals with HF. As expected, HF induced a reduction in endothelial-dependent dilation (EDD), as evidenced by the decrease in the EDD-Ach curve compared to the control group (Figs. 1A, 1B). Dapagliflozin treatment significantly increased EDD (Fig. 1A), thereby leading to an increase in the AUC of EDD (Fig. 1B). Endothelial dysfunction is also associated with cytokines imbalance in the serum. Thus, we measured the levels of common cytokines, including MCP-1, IL-1β, IL-6, and IL-17, which contribute to inflammation in HF. The levels of MCP-1, IL-1β, IL-6, and IL-17 were elevated in the HF group compared to the control group (Figs. 1C–1F). However, dapagliflozin treatment significantly reduced the levels of these cytokines (Figs. 1C–1F), indicating its potential in inhibiting inflammation in HF. Collectively, our findings suggest that dapagliflozin treatment may alter endothelial dysfunction in HF.

### Dapagliflozin ameliorated cardiac hypertrophy and dysfunction in HF

**Figure 2:**
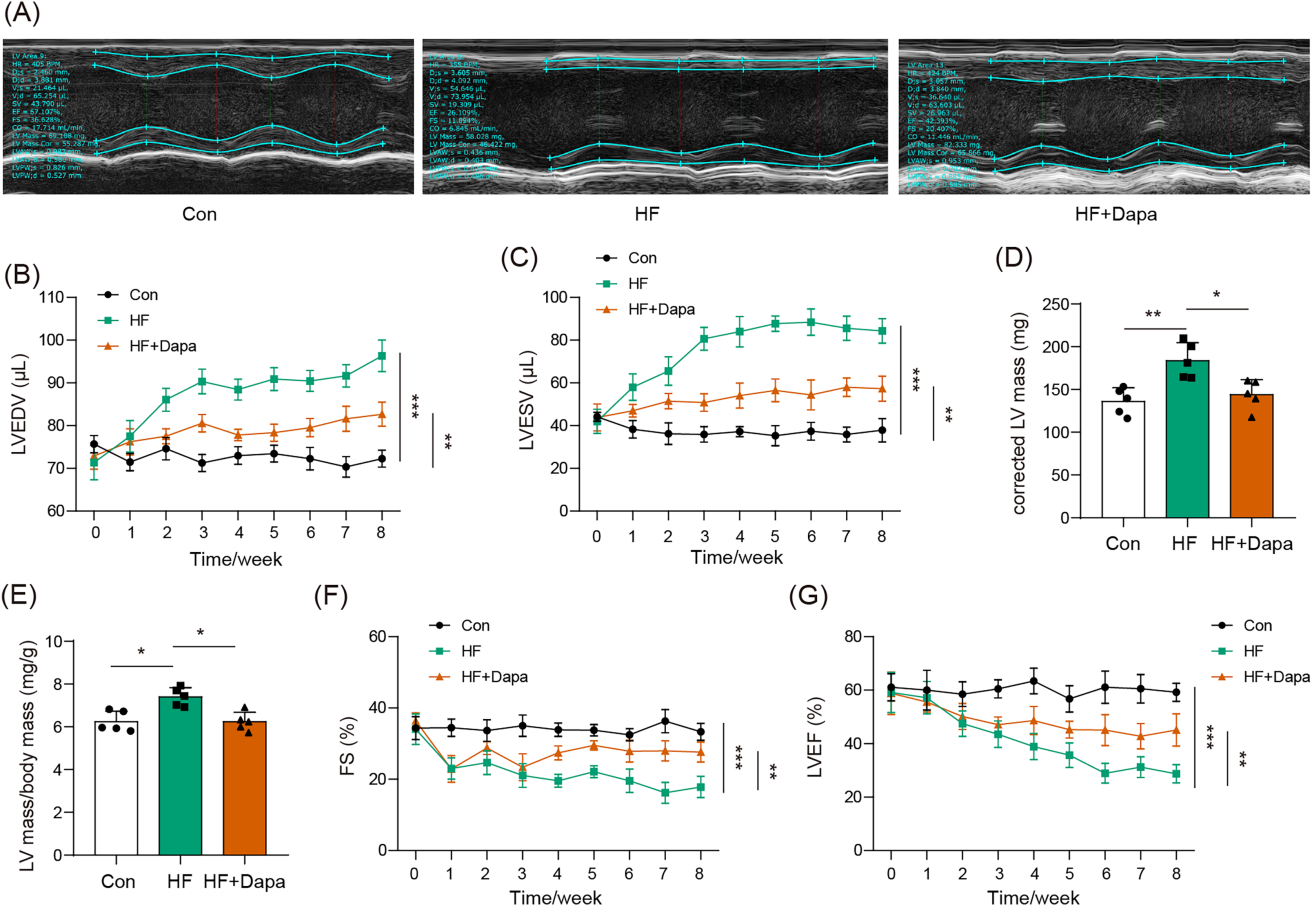
Dapagliflozin ameliorated cardiac hypertrophy and dysfunction in HF (*n* = 6). *p < 0.05, **p < 0.01 and ***p < 0.001.

Our study found that C57BL/6N mice with HF experienced significant cardiac hypertrophy, which was positively impacted by dapagliflozin treatment (Fig. 2A). We measured indices of cardiac function to evaluate the efficacy of dapagliflozin in treating HF *in vivo*. In the HF group, we observed a significant increase in left ventricular end-diastolic volume (LVEDV) (Fig. 2B), left ventricular end-systolic volume (LVESV) (Fig. 2C), LVEDV (Fig. 2B), LVESV (Fig. 2C), left ventricle (LV) mass (Fig. 2D), and the ratio of LV mass/body mass (Fig. 2E), along with a clear reduction in fractional shortening (FS) (Fig. 2F) and left ventricular ejection fraction (LVEF) (Fig. 2G), indicating cardiac decompensation. Notably, dapagliflozin treatment reversed these abnormal changes in LVEDV, LVESV, LV mass, the ratio of LV mass/body mass, FS, and LVEF in the HF group (Figs. 2B–2G). Additionally, mice in the HF group showed a decreased heart rate, while dapagliflozin increased the heart rate of mice with HF (Fig. S7). Figure S8 showed the change in body weight at the last week. Mice in HF group developed the increased body weight as compared to mice in control group. However, dapagliflozin treatment could reduce body weight of mice with HF.

### Myocardial infarct was inhibited by dapagliflozin treatment in HF progression

**Figure 3:**
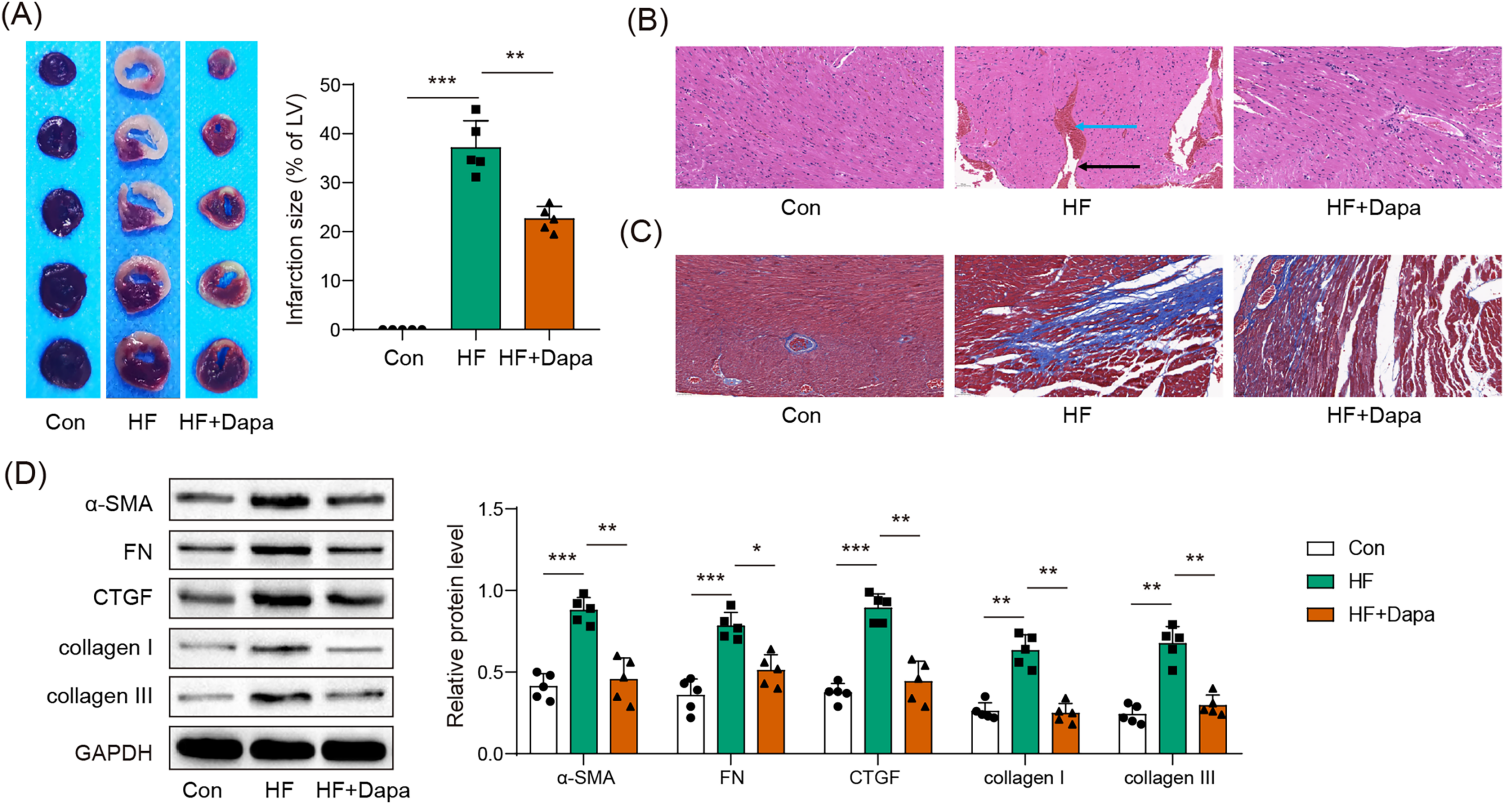
Myocardial infarct were inhibited by dapagliflozin treatment in HF (*n* = 6). *p < 0.05, **p < 0.01 and ***p < 0.001.

HF is often accompanied by cardiac ischemia and myocardial infarction. We investigated whether dapagliflozin could reduce the severity of myocardial infarction. We observed an increase in the infarct area in the HF group compared to healthy animals (Fig. 3A), accompanied by a disarray of cardiomyocytes (HF group in Fig. 3B: black arrow) and infiltration of inflammatory cells (HF group in Fig. 3B: blue arrow), which was ameliorated by dapagliflozin treatment (HF+Dapa group in Fig. 3B). In addition, HF induced interstitial fibrosis with collagen fibers deposition (HF group in Fig. 3B: dyed blue part) around the myocardial tissue, and dapagliflozin reduced the collagen fibers in HF (Fig. 3C). We measured molecular markers of myocardial fibrosis to assess myocardial injury at the molecular level. Our results showed that dapagliflozin treatment decreased HF-induced expressions of α-SMA, FN, collagen I/III, and CTGF in myocardial tissue (Fig. 3D). Therefore, we demonstrated that deposition inhibited myocardial infarction and cardiac fibrosis in HF progression.

### Community composition of fecal microbiota in dapagliflozin-treated HF

**Figure 4:**
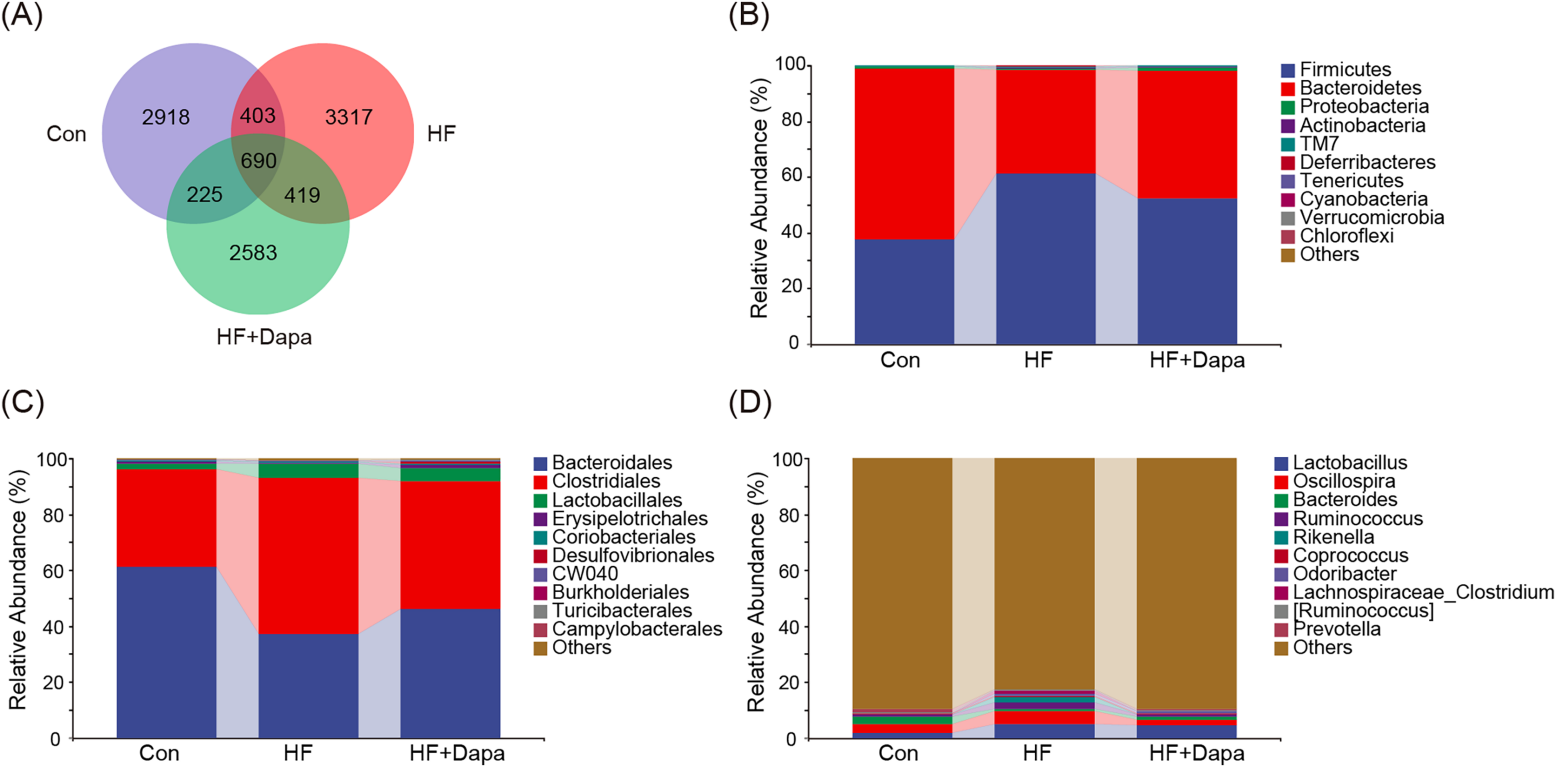
Community composition of fecal microbiota in dapagliflozin-treated HF (*n* = 6).

The gut microbiota play a central role in the progression of HF, and alterations in gut microbiota are important for understanding the pathogenesis of the disease. We collected fecal samples to analyze the microbiome using 16S rDNA sequencing. We found that the number of operational taxonomic units (OTUs) in the HF group was significantly increased to 3,317 compared to the control group and that dapagliflozin treatment decreased the number of OTUs (Fig. 4A). The two major phyla were *Firmicutes* and *Bacteroidetes* (Fig. 4B), and we calculated the ratio of Firmicutes/Bacteroidetes (F/B) as a marker of gut dysbiosis during disease progression. Notably, dapagliflozin treatment decreased the HF-induced increase in the F/B ratio. At the order level, *Bacteroidales* and *Clostridiales* were the two orders of gut microbiota (Fig. 4C), while *Lactobacillus, Oscillospira, Bacteroides*, and *Ruminococcus* were the four major genera at the genus level (Fig. 4D).

### Dapagliflozin-induced alterations in gut microbiota based on α- and ${\bf{\beta }}$- diversity

**Figure 5:**
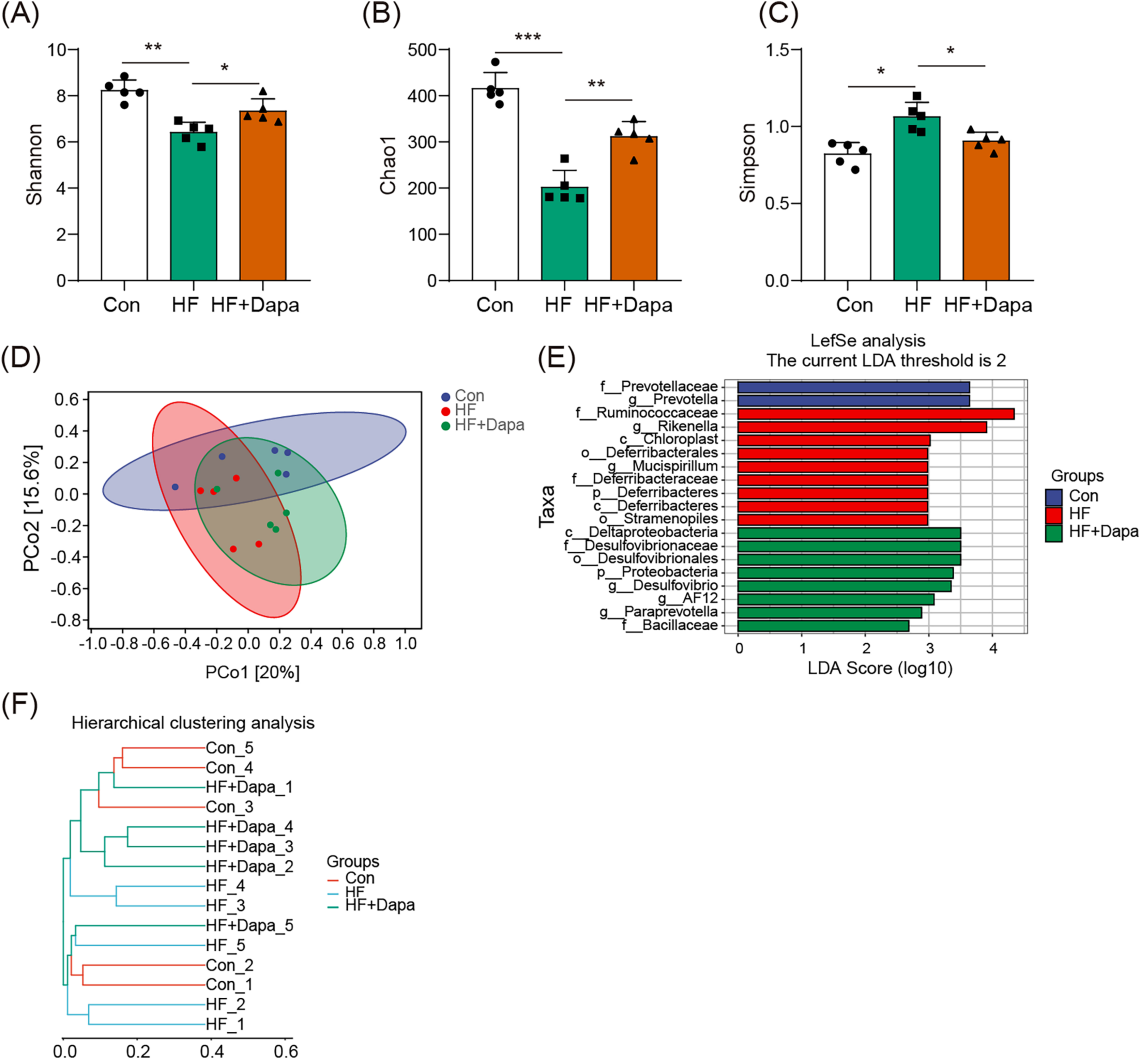
Dapagliflozin-induced alterations in gut microbiota based on a- and b-diversity (*n* = 6). *p < 0.05, **p < 0.01 and ***p < 0.001.

We investigated the diversity of gut microbiota in dapagliflozin-treated HF using Chao1, Shannon and Simpson indices as a measure of within-habitat diversity (α-diversity). The HF group exhibited a significant decrease in Shannon (Fig. 5A) and Chao1 (Fig. 5B) indices, but an increase in Simpson index (Fig. 5C) compared to the control group; dapagliflozin treatment increased Shannon and Chao1 indices while reducing Simpson index in the gut microbiota of mice with HF, with statistical significance observed by Kruskal–Wallis test. We calculated sample distance using Bray-Curtis, unweighted UniFrac, and weighted UniFrac methods and performed dimensionality reduction using principal coordinate analysis (PCoA) and non-metric multidimensional scaling (NMDS). There was a clear distance among control, HF, and HF+dapagliflozin groups according to PCoA (Fig. 5D, Fig. S2) and NMDS (Fig. S3), suggesting the alteration of gut microbiota communities in the three groups. At the genus level, we found that *Desulfovibrio*, *AF12*, and *Paraprevotella* were enriched in the HF+dapagliflozin group, while *Rikenella* and *Mucispirillum* could be potential biomarkers of the HF group (Fig. 5E). Hierarchical cluster analysis demonstrated that the HF+dapagliflozin group exhibited greater consistency compared to the other groups (Fig. 5G).

### Functional prediction of altered gut microbiota in dapagliflozin-treated HF progression

**Figure 6:**
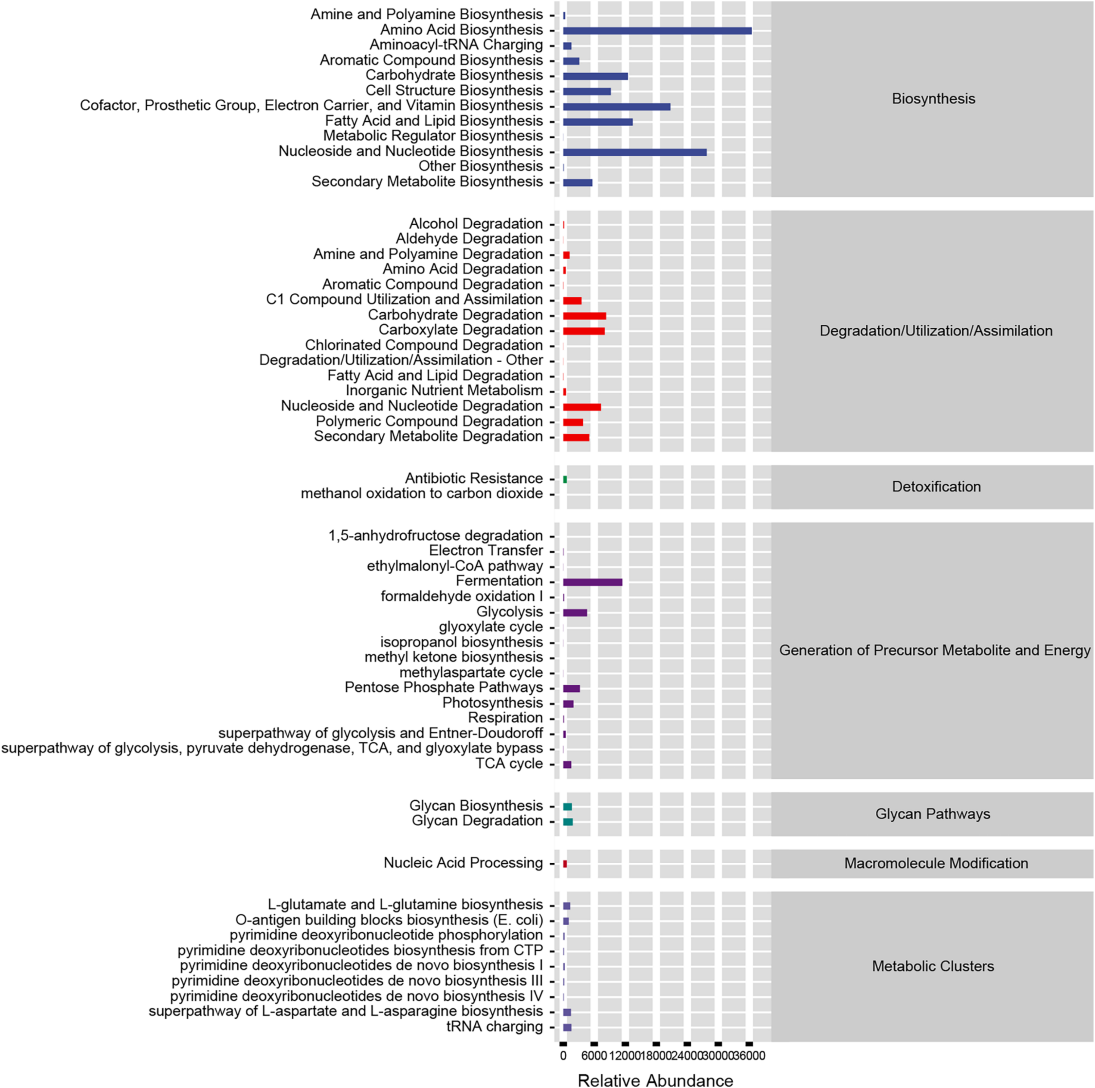
Functional prediction of altered gut microbiota.

Based on our analysis using the KEGG database, we identified that altered gut microbiotas were associated with various metabolic pathways, including fermentation, amino acid biosynthesis, nucleoside and nucleotide biosynthesis/degradation, fatty acid and lipid biosynthesis, carbohydrate biosynthesis/degradation, cofactor/prosthetic group/electron carrier/vitamin biosynthesis (Fig. 6). To further understand the potential implication of these alterations, we screened and identified the significantly altered pathways that were correlated to the altered microbiotas. Among these, biotin biosynthesis II, photorespiration, super pathway of sulfolactate degradation, and superpathway of taurine degradation were significantly upregulated (Supplemental Materials). These findings suggest that the gut microbiota may play a key role in regulating various metabolic processes that contribute to the progression of HF.

### Validation of the microbiota-mediated role of dapagliflozin in HF based on fecal microbiota transplantation (FMT) in germ-free mice

To validate whether gut microbiota plays a role in HF onset, we performed an FMT experiment using germ-free mice. FMT was conducted using fecal samples from mice with HF or healthy donors, and some of the recipients with HF were treated with dapagliflozin. As shown in Fig. S9, FMT from HF donors impaired endothelial function and induced inflammation in recipient mice, and these efforts were improved by dapagliflozin treatment. Figure S10 suggests that the recipients developed decreased cardiac motion amplitude due to HF microbiota, which was increased by dapagliflozin treatment. In terms of histopathology, the increased area of myocardial infarction was observed in HF recipients (Fig. S11A); also, these recipients developed inflammation infiltration into myocardial tissue (Fig. S11B) and myocardial fibrosis (Fig. S11C). At the molecular level, the expression of α-SMA, FN, CTGF, collagen I, and collagen III were upregulated in recipients with HF (Fig. S11D). However, dapagliflozin treatment reversed these changes in Fig. S11 due to HF microbiota in recipients.

## Discussion

Heart failure (HF) is one of the major reasons of mortality worldwide. Dapagliflozin has the potential protective role in HF by its ability to modulate the gut microbiota composition. However, the definite underlying mechanism of the dapagliflozin-gut-heart axis is not understood. The results of this investigation have demonstrated that treatment with dapagliflozin can improve HF progression in a C57BL/6N mouse model by resisting endothelial dysfunction. More importantly, we screened the microbiota profile of dapagliflozin-treated HF mice. *AF12* was found to be the therapeutic target of dapagliflozin at the genus level, and we also identified *Rikenella* and *Mucispirillum* as potential biomarkers of HF.

Withaar et al. (2021) have demonstrated that dapagliflozin can regulate glucose metabolism and partly affect cardiac function in the model of heart failure with preserved ejection fraction. Similarly, our results suggested that dapagliflozin had a myocardial-protective role in HF progression due to a reduction in hypertrophic myocardium, inhibition of myocardial fibrosis, and decreased area of myocardial infarction. Endothelial dysfunction is the poor cardiovascular event in HF. Previous evidence (Lee et al., 2018) has highlighted the protective role of dapagliflozin in endothelial function in cardiovascular diseases. We further observed that dapagliflozin reversed EDD injury in mice with HF, and endothelial dysfunction is involved in inflammation, immune response, and cardiac function. On the one hand, endothelial barrier impairment results in serum cytokine disorder contributing to local or systematic inflammation in the body; while alteration in EDD leads to systolic and diastolic dysfunction (Zuchi et al., 2020). Collectively, dapagliflozin can treat HF progression *via* the improvement of endothelial dysfunction in addition to the modulation of SGLT2-involved glycemic control and other kinds of metabolism.

Gut microbiota serves as a contributing factor in HF progression. We observed a significant alteration in the composition of gut microbiota among control, HF, and HF+dapagliflozin groups. Based on LEfSe analysis, we found *AF12*, *Paraprevotella*, and *Desulfovibrio* to be significantly enriched in the HF+dapagliflozin group, and *Rikenella* and *Mucispirillum* to be elevated in the HF group. We described *AF12* as therapeutic targets of dapagliflozin, and *Rikenella* and *Mucispirillum* were defined as HF biomarkers. Current evidence (Ghane et al., 2013; Hanninen et al., 2018) suggests that *AF12* genus delays diabetes progression and resists heavy metal damage. Dapagliflozin can elevate *AF12* abundance by inhibiting poor cardiovascular events in HF progression. However, dapagliflozin simultaneously evokes potential side effects. *Paraprevotella* has been defined as a significant bacteria contributing to damage to cardiac structure and function in a rat model of HF (Gutierrez-Calabres et al., 2020). *Desulfovibrio*, as a harmful genus, is the core member of sulfate-reducing bacteria in gut microbiota that lead hydrogen sulfide and lipopolysaccharide into impaired tissue (Murros et al., 2021). The enrichment of *Paraprevotella* and *Desulfovibrio* may be an adverse event during dapagliflozin treatment. We infer AF12-involved protection may, fortunately, offset the injury caused by *Paraprevotella* and *Desulfovibrio* in HF mice during dapagliflozin treatment, which is one of the significant improvements of mice with HF during dapagliflozin treatment. *Rikenella* and *Mucispirillum* were also increased in mice with HF. *Rikenella* is an important genus involved in lipid metabolism and glycometabolism with varied abundance in different models (Hu et al., 2021; Zhou et al., 2018). HF is a complex heterogeneous disease in which *Rikenella* may induce a serious disorder of lipid and glucose that lead to a physiological burden in the heart and blood vessels. *Mucispirillum’*s abundance in HF is related to pathogenesis heterogeneity. A model of hypertension-induced HF was characterized by the decrease of *Mucispirillum* as per a previous report (Gutierrez-Calabres et al., 2020), whereas its abundance was elevated when the HF model was construed by myocardial ischemia in our findings. The abundance of *Mucispirillum* plays a pathogenesis-specific role in HF. Also, we found germ-free mice undergoing FMT developed impairment of endothelial function and myocardial function, suggesting the significant role of gut microbiota in HF.

Recent study by Li et al. (2023) showed the role of dapagliflozin in gut microbiota based on a model of LDA ligation. They found dapagliflozin modulated gut microbiota to improve myocardial dysfunction. Our findings demonstrated dapagliflozin could improve endothelial dysfunction in mice undergoing HF *via* targeting gut microbiota, thereby contributing to the recovery of myocardial function. It is the difference between our findings and the report by Li et al. (2023). The functional prediction of altered microbiota indicated these microbiotas ameliorated endothelial dysfunction in mice with HF by modulating the biosynthesis and degradation of metabolites. The potential functional relationship between altered gut microbiota and the metabolism of amino acid, fatty acid, and lipid, carbohydrate, and vitamins has now been established. Changes in metabolism function as the physiological basis of the cardiac disorder (Wang & Zhao, 2018).

Meanwhile, the gut microbiota is the core controller of metabolites in the human body. For example, *Paraprevotella* elevated by dapagliflozin mediates vitamin synthesis and carbohydrate degradation, thereby affecting glycemic control and metabolic homeostasis (Lu et al., 2021). Thus, dapagliflozin regulates the composition of gut microbiota to resist adverse events in HF progression. Microbiota-induced changes in these metabolites will participate in inflammation and endothelial dysfunction that modulate cardiac overload and injury in HF progression (Eelen et al., 2015; He et al., 2020; Kim et al., 2020). Moreover, among control, HF, and HF+dapagliflozin groups, biotin biosynthesis II, photorespiration, superpathway of sulfolactate degradation, and superpathway of taurine degradation were significantly upregulated due to the alteration of gut microbiota. Biotin, sulfolactate, and taurine metabolism can develop the progression of cardiovascular disease. As known, biotin is an indispensable component of metabolic activities (Mock, 2017). Sulfolactate plays an acritical role in lipid metabolism and the TCA cycle (MetaCyc, 2022). Taurine has been demonstrated to regulate Ca^2+^ homeostasis and antioxidant in cardiovascular adverse events (Bkaily et al., 2020). Potentially, microbiota-induced metabolic change is the reason why the dapagliflozin-gut-heart axis can play a significant role during HF. Fecal microbiota transplantation also demonstrated the ability of dapagliflozin to reverse microbiota-induced endothelial and myocardial dysfunction. Collectively, our findings provide the different therapeutic mechanisms of dapagliflozin at the microbiological level in addition to the regulation of the expression and activity of SGLT2.

## Conclusions

One limitation of the present investigation is the lack of a diagnostic model to validate the risk assessment value of microbial markers, which will be addressed in follow-up studies. In summary, this study provides insights into the microbiome profile of a mouse model of HF and sheds light on the therapeutic mechanism of dapagliflozin for HF. Dapagliflozin has the potential capability to improve endothelial dysfunction and myocardial infarction during heart failure. Moreover, the identified signature microbiota may offer a convenient and cost-effective method for distinguishing between HF patients and healthy individuals.

## Supplemental Information

10.7717/peerj.15589/supp-1 Supplemental Information 1 Differential analysis of metabolic pathways.

10.7717/peerj.15589/supp-2 Supplemental Information 2 PCoA result based on unweighted_unifrac distance and weighted_unifrac distance.

10.7717/peerj.15589/supp-3 Supplemental Information 3 NMDS results based on Bray_Crutis distance, unweighted_unifrac distance and weighted_unifrac distance.

10.7717/peerj.15589/supp-4 Supplemental Information 4 The rarefaction curve.

10.7717/peerj.15589/supp-5 Supplemental Information 5 The relative abundance of biomarkers according to LEfSe.

10.7717/peerj.15589/supp-6 Supplemental Information 6 Difference analysis based on PERMANOVA and ANOSIM.

10.7717/peerj.15589/supp-7 Supplemental Information 7 Dapagliflozin increased heart rate of mice with HF.

10.7717/peerj.15589/supp-8 Supplemental Information 8 Dapagliflozin reduced the body weight of mice with HF.

10.7717/peerj.15589/supp-9 Supplemental Information 9 Dapagliflozin improved endothelial function of mice transplanted with fecal microbiota from HF mice.

10.7717/peerj.15589/supp-10 Supplemental Information 10 Dapagliflozin reversed the impaired cardiac motion amplitude due to HF microbiota.

10.7717/peerj.15589/supp-11 Supplemental Information 11 Dapagliflozin inhibited HF microbiota-induced myocardial infarction.

10.7717/peerj.15589/supp-12 Supplemental Information 12 Raw measurements for Figure 1.

10.7717/peerj.15589/supp-13 Supplemental Information 13 Raw measurements for Figure 2.

10.7717/peerj.15589/supp-14 Supplemental Information 14 Raw measurements for Figure 3.

10.7717/peerj.15589/supp-15 Supplemental Information 15 Western blot for Figure 3D.

10.7717/peerj.15589/supp-16 Supplemental Information 16 Author Checklist.

10.7717/peerj.15589/supp-17 Supplemental Information 17 All the experimental conditions, number of animals, and mean, SEM, and *p*-value for all the three repeats for each experiment.

10.7717/peerj.15589/supp-18 Supplemental Information 18 Raw sequence data of samples in control group, including con_1, con_2 and con_3. con_1-R1: ZTPSN21HD929-1_1_R1; con_1-R2: ZTPSN21HD929-1_1_R2.; con_2-R1: ZTPSN21HD930-1_2; con_2-R2: ZTPSN21HD930-1_2_R2; con_3-R1: ZTPSN21HD931-1_3_R1; con_3-R2: ZTPSN21HD931-1_3_R2

10.7717/peerj.15589/supp-19 Supplemental Information 19 Raw sequence data of samples in control group (con_4 and con_5) and HF group (HF_1). con_4-R1: ZTPSN21HD932-1_4_R1; con_4-R2: ZTPSN21HD932-1_4_R2.; con_5-R1: ZTPSN21HD933-1_5_R1; con_5-R2: ZTPSN21HD933-1_5_R2; HF_1-R1: ZTPSN21HD934-3_1_R1; HF_1-R2: ZTPSN21HD934-3_1_R2

10.7717/peerj.15589/supp-20 Supplemental Information 20 Raw sequence data of samples in HF group, including HF_2, HF_3 and HF_4. HF_2-R1: ZTPSN21HD935-3_2_R1; HF_2-R2: ZTPSN21HD935-3_2_R2; HF_3-R1: ZTPSN21HD936-3_3_R1; HF_3-R2: ZTPSN21HD936-3_3_R2; HF_4-R1: ZTPSN21HD937-3_4_R1; HF_4-R2: ZTPSN21HD937-3_4_R2

10.7717/peerj.15589/supp-21 Supplemental Information 21 Raw sequence data of samples in HF group (HF_5) and HF+Dapa group (HF_Dapa_1 and HF_Dapa_2). HF_5-R1: ZTPSN21HD938-3_5_R1; HF_5-R2: ZTPSN21HD938-3_5_R2; HF_Dapa_1-R1: ZTPSN21HD939-4_1_R1; HF_Dapa_1-R2: ZTPSN21HD939-4_1_R2; HF_Dapa_2-R1: ZTPSN21HD940-4_2_R1; HF_Dpa_2-R2: ZTPSN21HD940-4_2_R2

10.7717/peerj.15589/supp-22 Supplemental Information 22 Raw sequence data of samples in HF+Dapa group, including HF_Dapa_3, HF_Dapa_4 and HF_dapa_5. HF_Dapa_3-R1: ZTPSN21HD941-4_3_R1; HF_Dapa_3-R2: ZTPSN21HD941-4_3_R2; HF_Dapa_4-R1: ZTPSN21HD942-4_4_R1; HF_Dapa_4-R2: ZTPSN21HD942-4_4_R2; HF_dapa_5-R1: ZTPSN21HD943-4_5_R1; HF_dapa_5-R2: ZTPSN21HD943-4_5_R2

## List of abbreviations

HF: heart failure
H&E: hematoxylin and eosin staining
TTC: 2,3,5-Triphenyltetrazolium chloride
H&E: hematoxylin (H), and eosin (E)
PCoA: Principal coordinate analysis
NMDS: non-metric multidimensional scaling
LEfSe: Linear discriminant analysis coupled with effect size measurements
PICRUSt: Phylogenetic investigation of communities through reconstruction of unobserved states
KEGG: Kyoto Encyclopedia of Genes and Genomes
SGLT2: sodium-glucose cotransporter-2
LAD: left anterior descending coronary artery
LV: Left ventricular
LVEDV: left ventricular end-diastolic volume
LVESV: left ventricular end-systolic volume
FS: fractional shortening (FS)
LVEF: left ventricular ejection fraction
Ach: acetylcholine
MI: myocardial infarction
EDD: endothelial-dependent dilation

## Additional Information and Declarations

### DNA Deposition

The following information was supplied regarding the deposition of DNA sequences: The sequences in control, HF, HF+Dapa groups are available at SRA: SRR20072335 to SRR20072329.

### Data Availability

The following information was supplied regarding data availability: The raw measurements are available in the Supplemental Files.
