# Supplementary figures and images for: Dapagliflozin-affected endothelial dysfunction and altered gut microbiota in mice with heart failure
Source: PeerJ. 2023 Jul 26;11:e15589. doi: 10.7717/peerj.15589 (PMC10386824; doi:10.7717/peerj.15589)

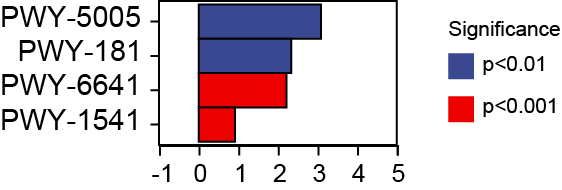

Supplement: Supplemental Information 1 [file peerj-11-15589-s001.png]

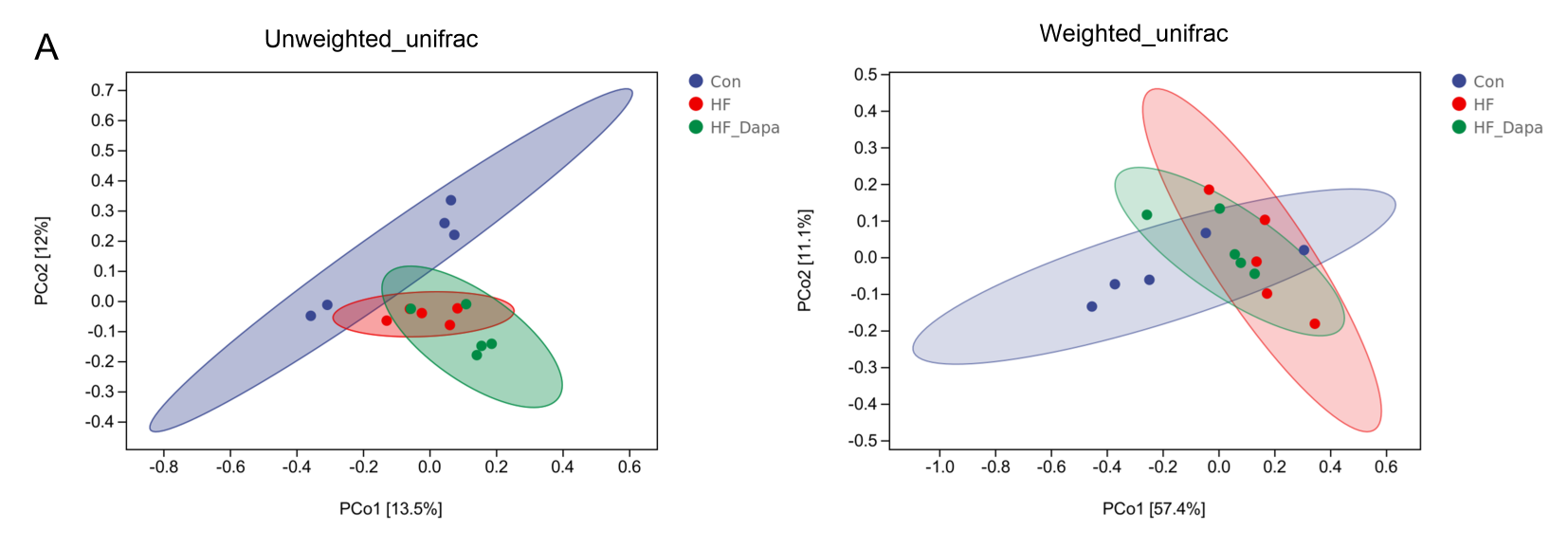

Supplement: Supplemental Information 2 [file peerj-11-15589-s002.png]

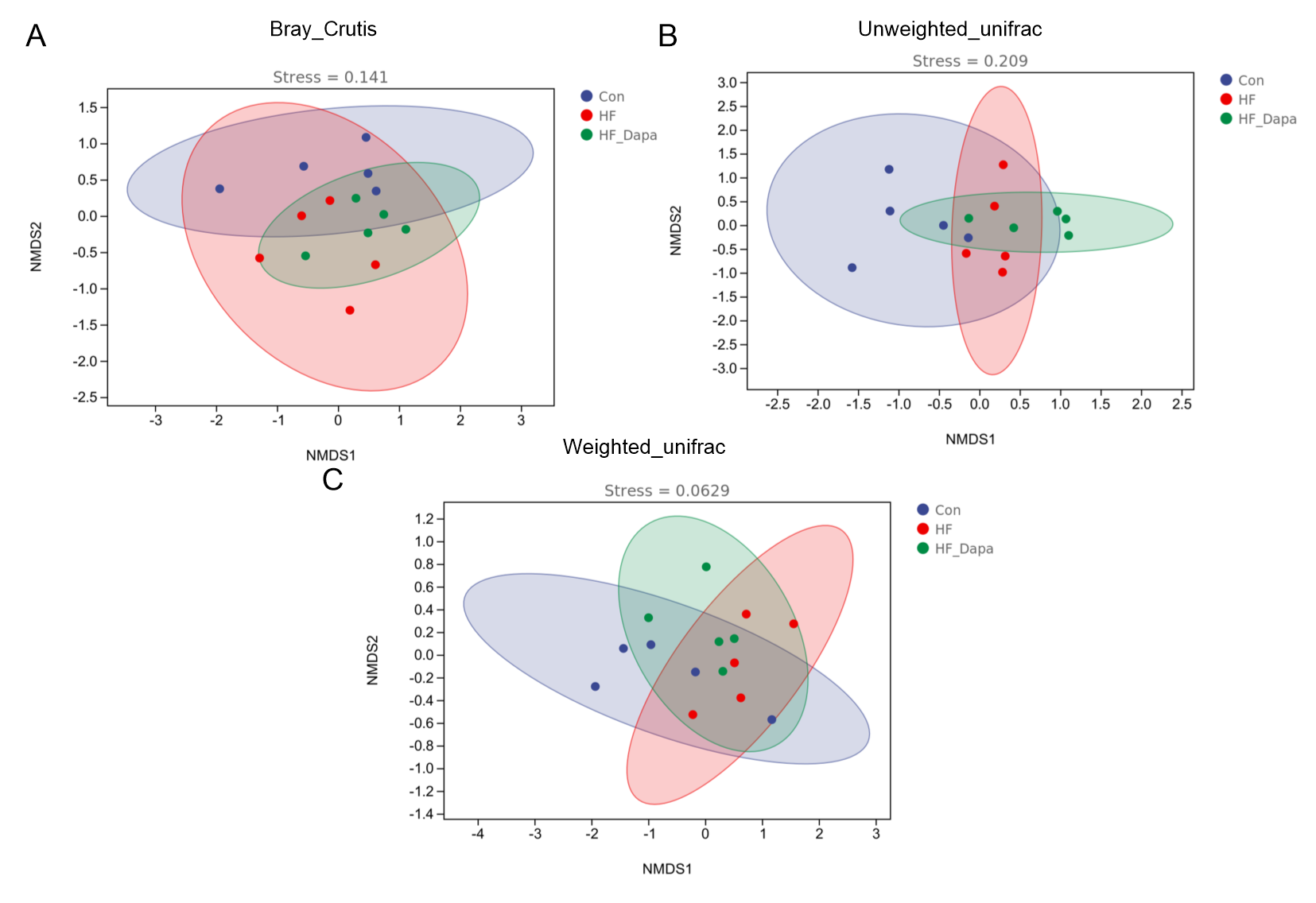

Supplement: Supplemental Information 3 [file peerj-11-15589-s003.png]

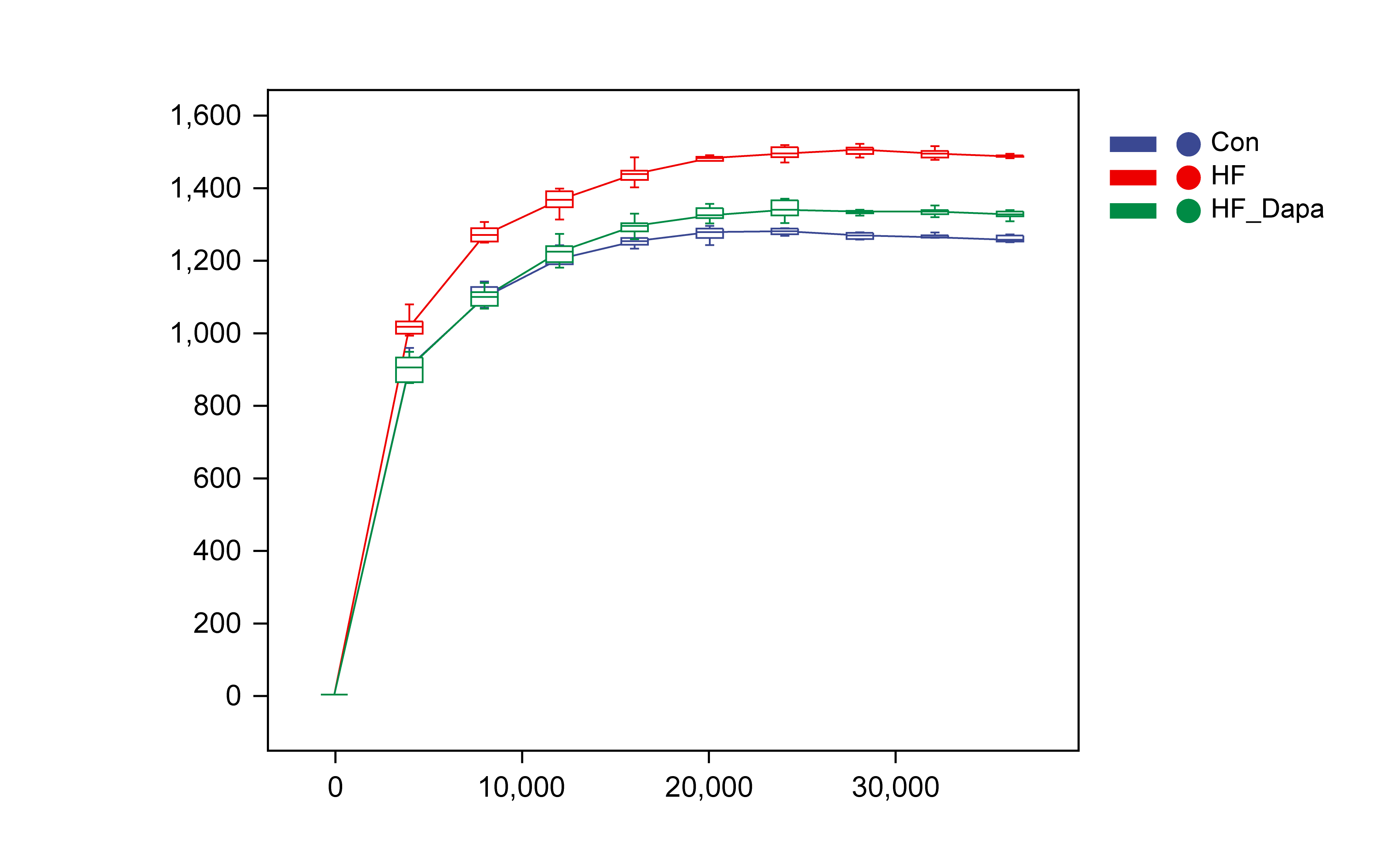

Supplement: Supplemental Information 4 [file peerj-11-15589-s004.png]

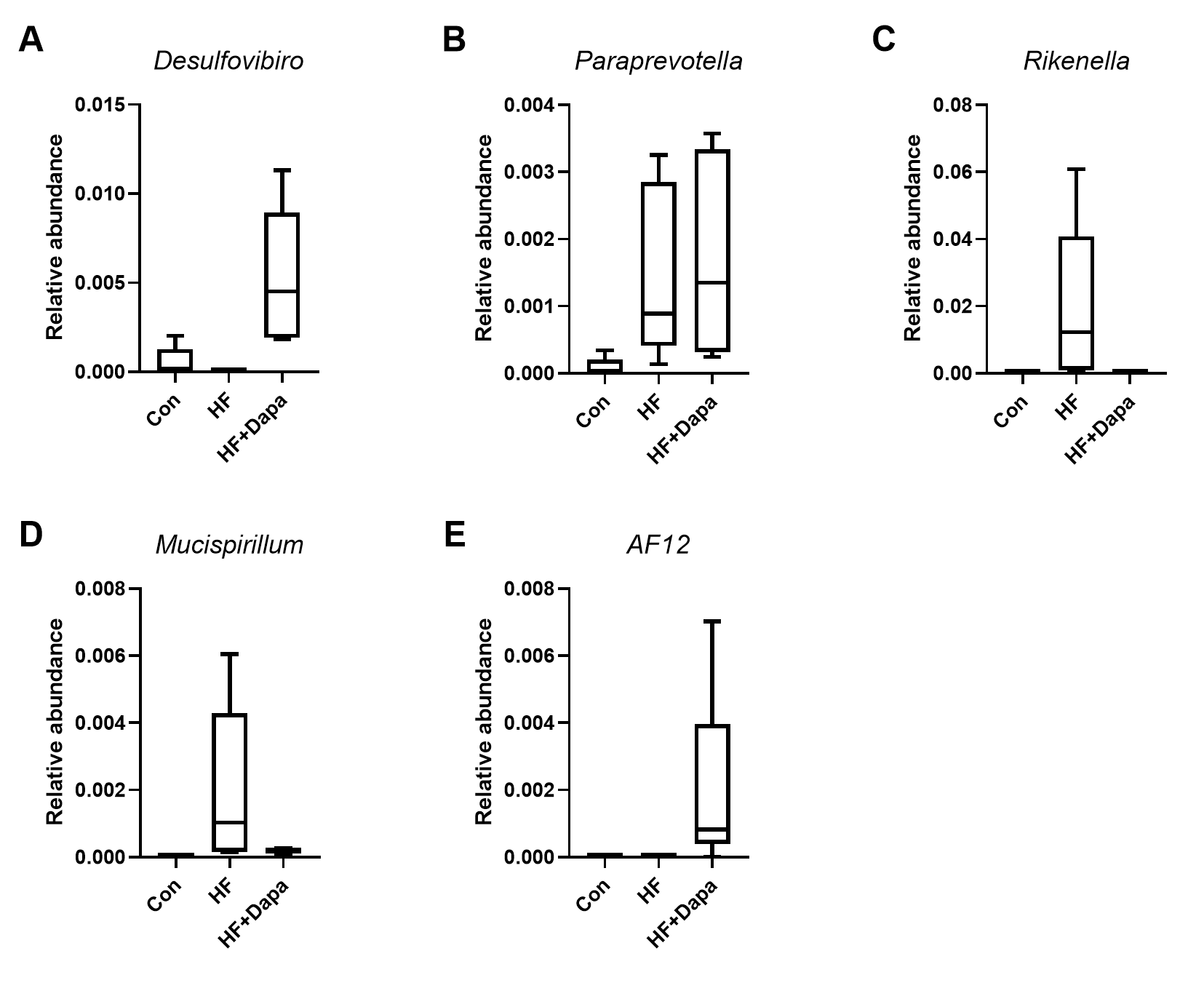

Supplement: Supplemental Information 5 [file peerj-11-15589-s005.png]

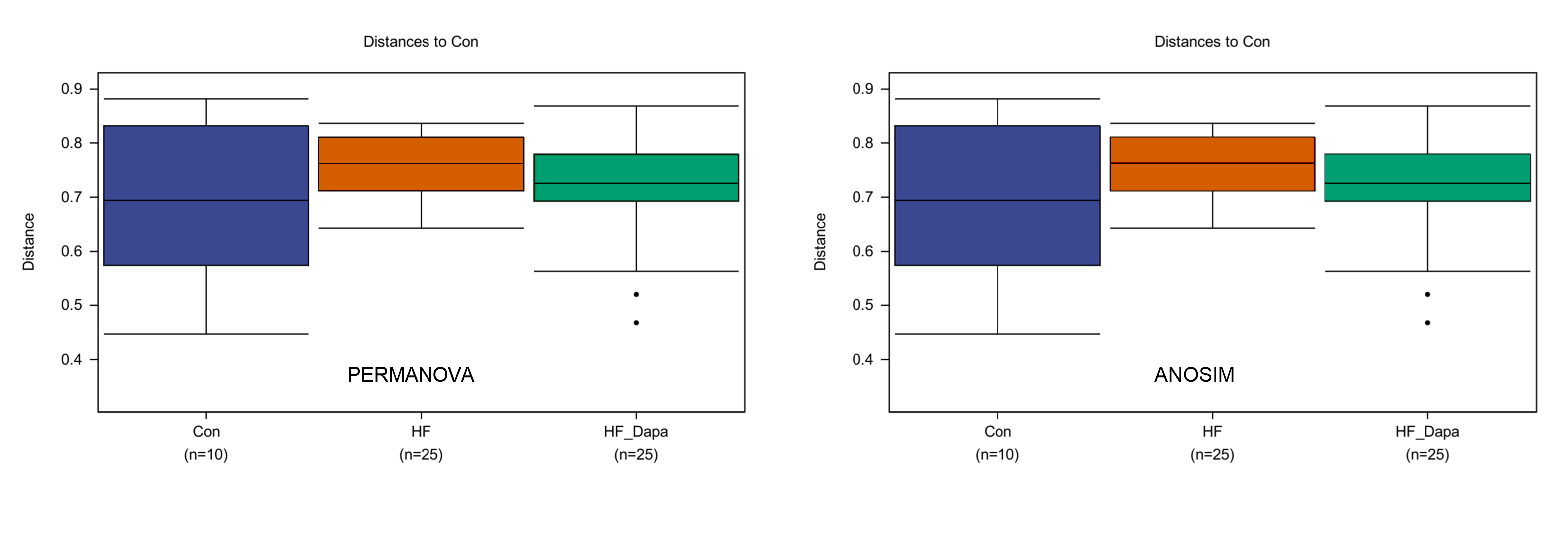

Supplement: Supplemental Information 6 [file peerj-11-15589-s006.png]

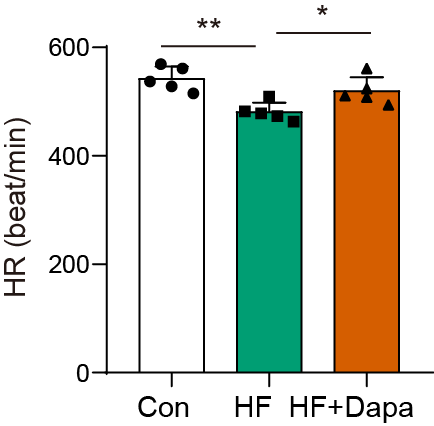

Supplement: Supplemental Information 7 [file peerj-11-15589-s007.png]

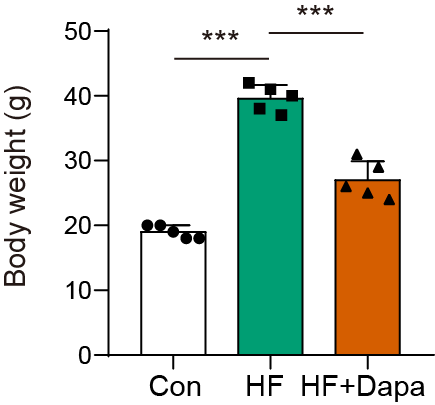

Supplement: Supplemental Information 8 [file peerj-11-15589-s008.png]

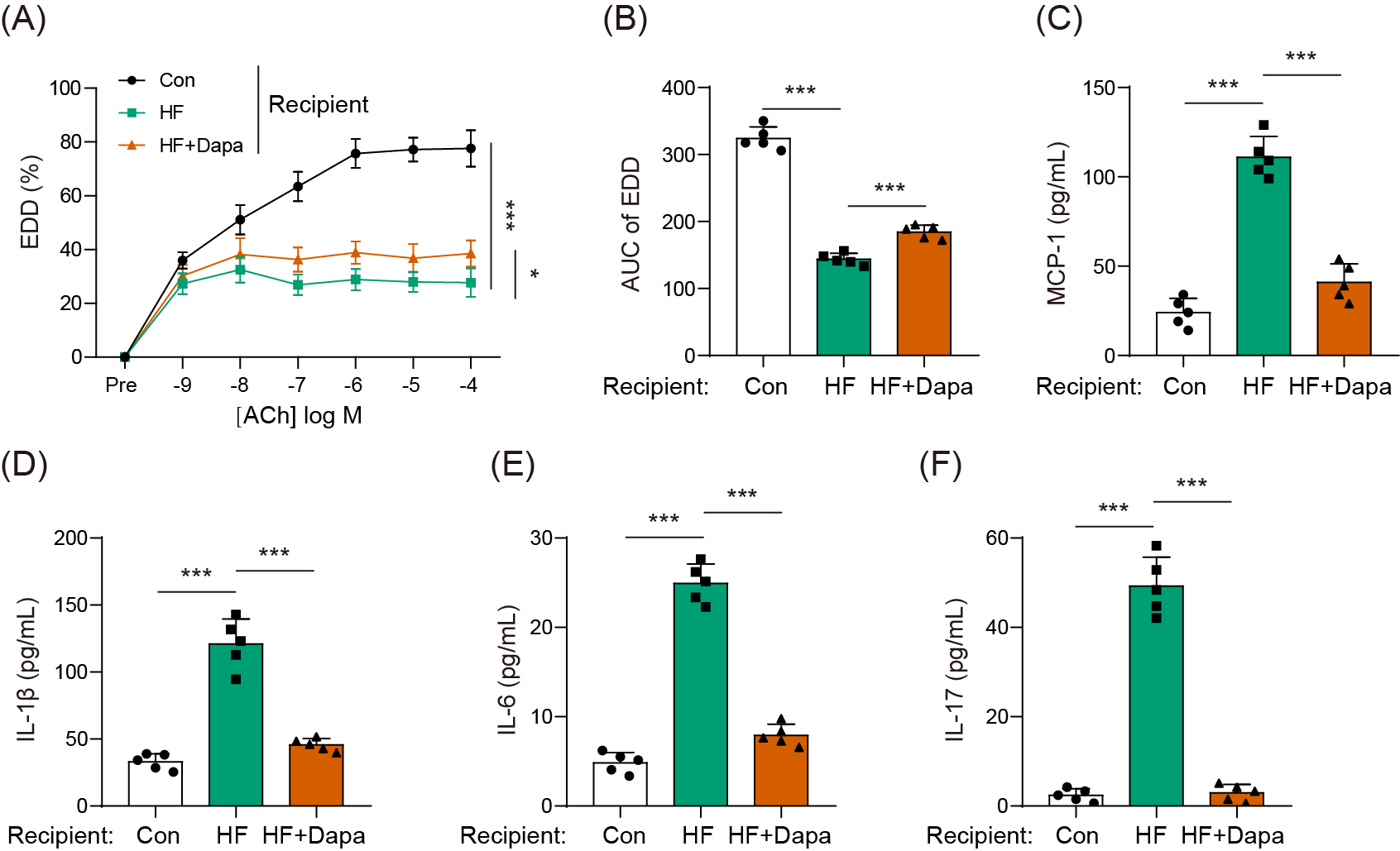

Supplement: Supplemental Information 9 [file peerj-11-15589-s009.png]

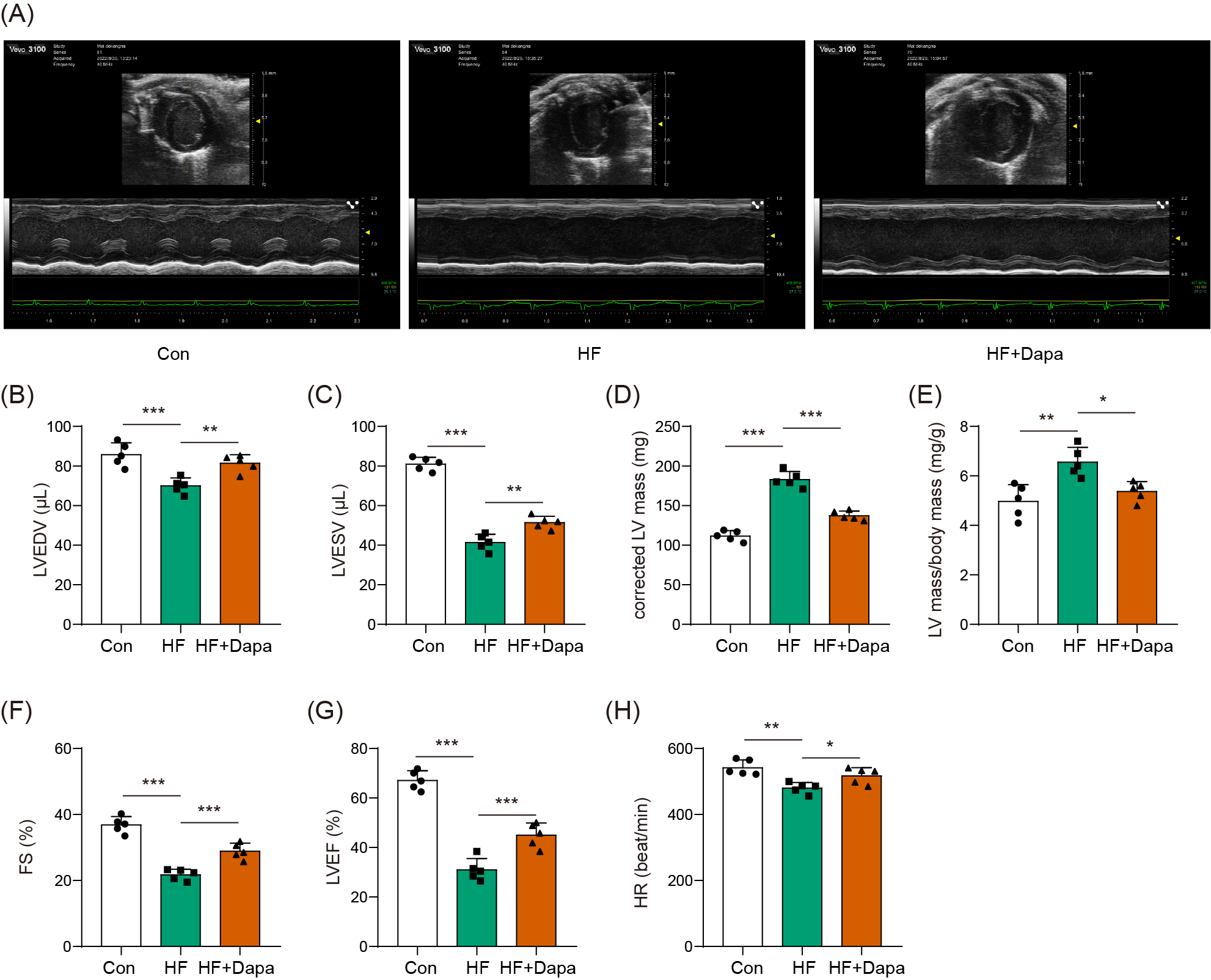

Supplement: Supplemental Information 10 [file peerj-11-15589-s010.png]

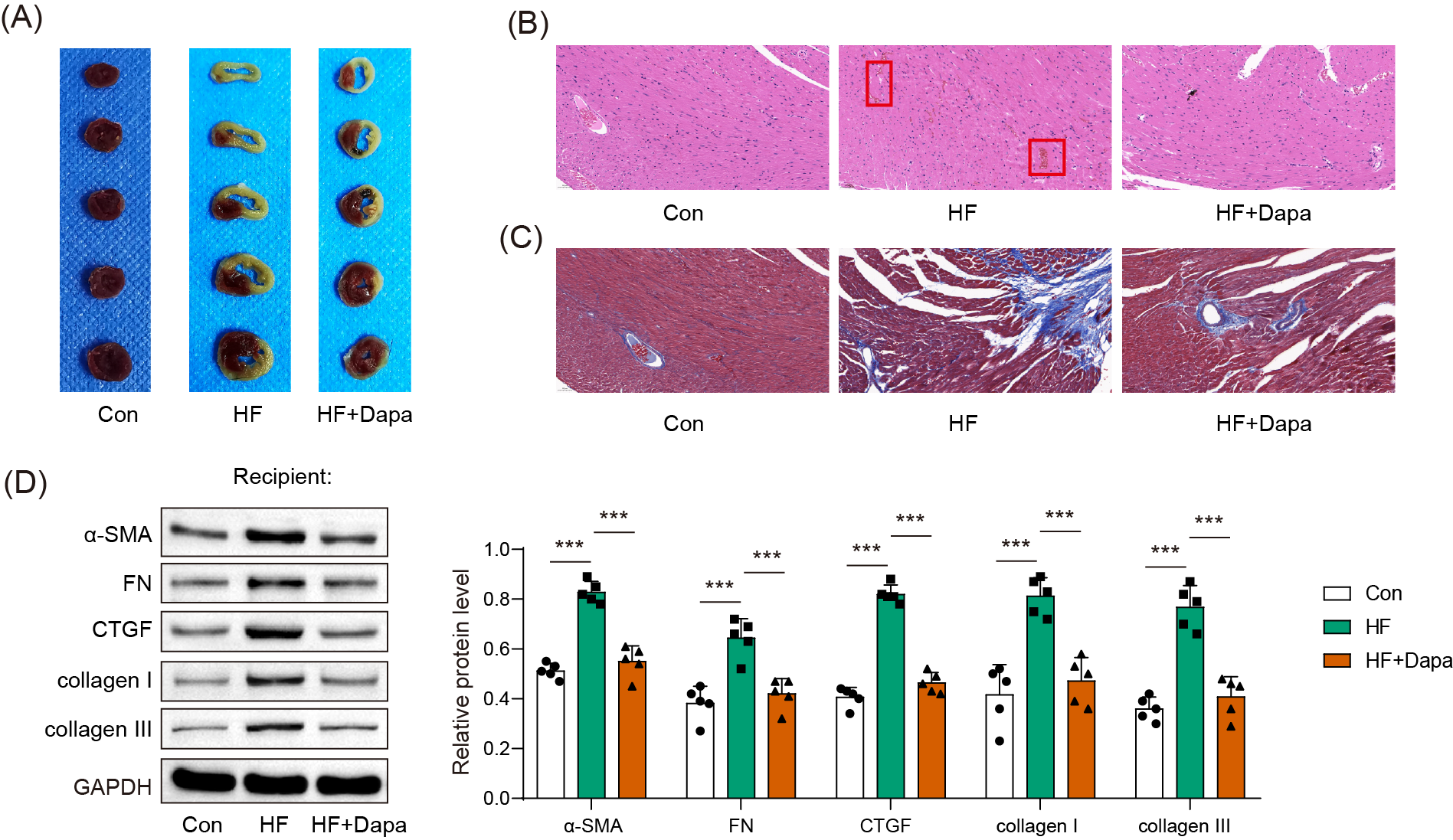

Supplement: Supplemental Information 11 [file peerj-11-15589-s011.png]

$\alpha$ -SMA

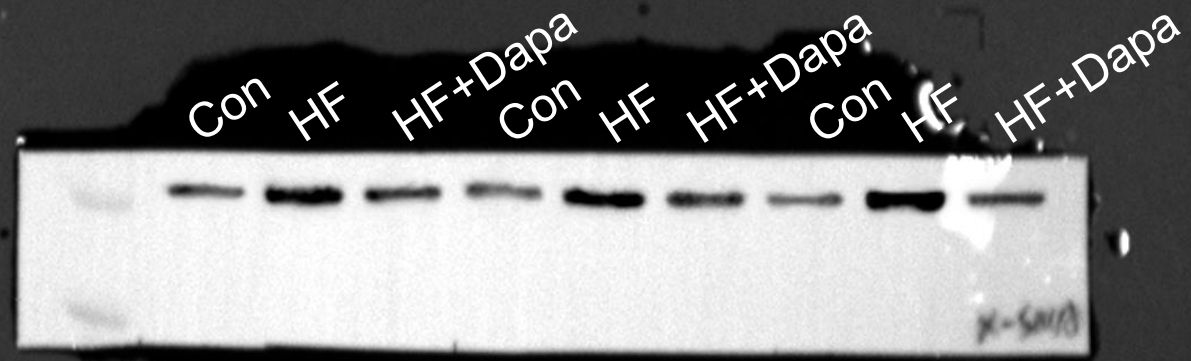

Collagen I

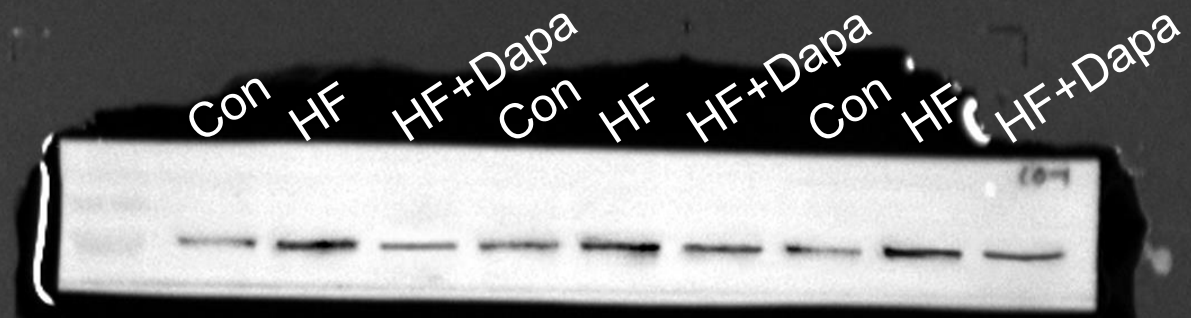

# Collagen III

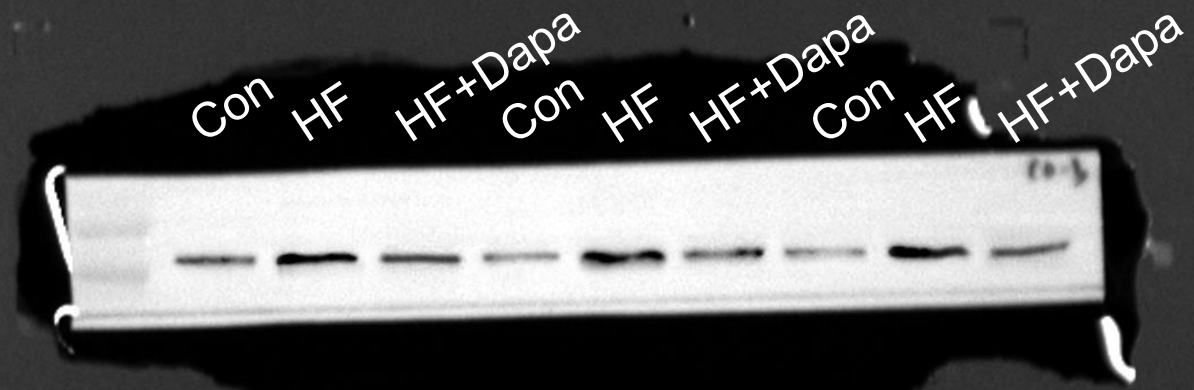

CTGF

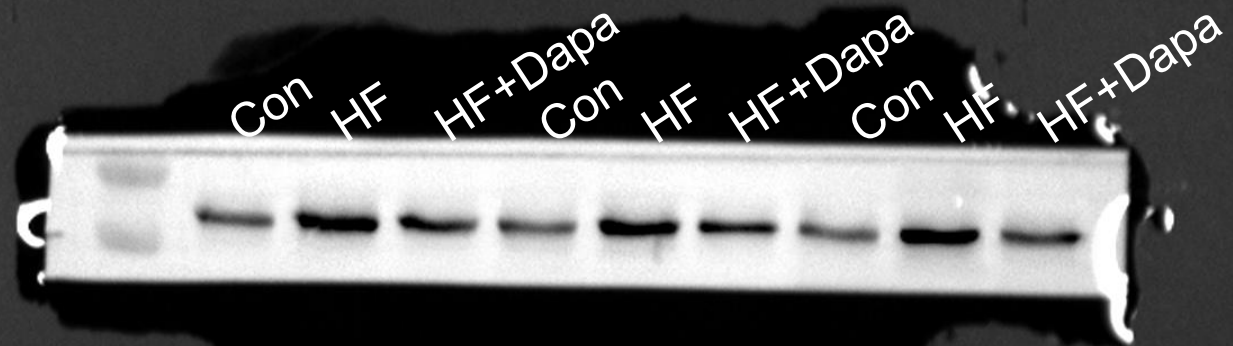

FN

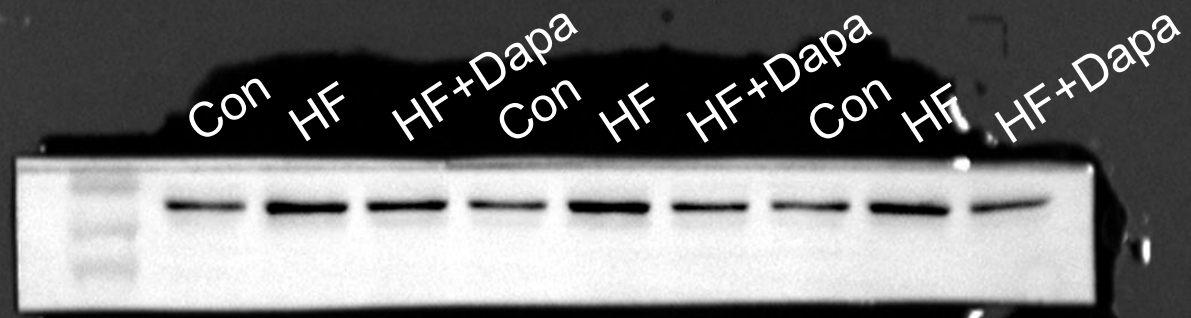

GAPDH

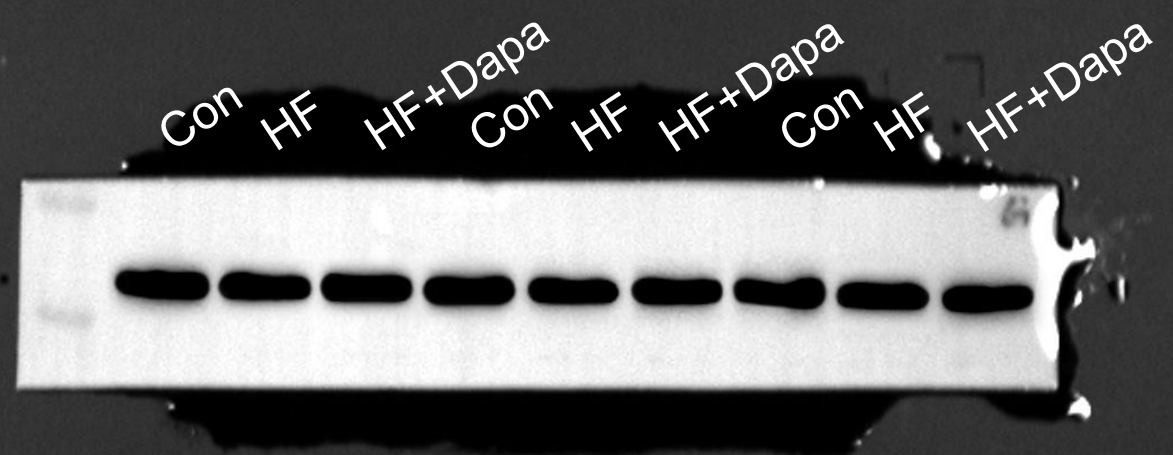

Supplement: Supplemental Information 15 [file peerj-11-15589-s015.pdf]
