# Supplementary material for: Dapagliflozin-affected endothelial dysfunction and altered gut microbiota in mice with heart failure
Source: PeerJ. 2023 Jul 26;11:e15589. doi: 10.7717/peerj.15589 (PMC10386824; doi:10.7717/peerj.15589)
Supplement: Supplemental Information 17 [file peerj-11-15589-s017.docx]

| Experiment | Experiment condition | Number of animals | Mean±SEM | P value |
| --- | --- | --- | --- | --- |
| AUC of EDD | a) control group, mice received thoracotomy without the ligation at left anterior descending coronary artery (LAD) after anesthetized with 100% oxygen and 1-4% isoflurane at 0.5 L/min;  b) HF group, mice received thoracotomy with the permanent ligation of of 6-0 nonabsorbable silk sutures at LAD for 8 weeks as previously described after anesthetized;  c) HF+dapagliflozin group intraperitoneally injected with dapagliflozin (1 mg/kg/day, until the end of the 8-week period) after LAD ligation. | n=5 in each group | Con:  318.9±20.70  HF:  95.43±15.20  HF+Dapa:  165.5±21.46 | Con vs HF: p<0.001  HF vs HF+Dapa: p<0.05 |
| Serum MCP-1 (pg/mL) |  |  | Con: 15.76±3.18  HF: 120.69±23.01  HF+Dapa:  25.17±6.23 | Con vs HF: p<0.001  HF vs HF+Dapa: p<0.001 |
| Serum IL-1β |  |  | Con: 18.11±3.06  HF: 153.87±24.91  HF+Dapa:  41.68±13.51 | Con vs HF: p<0.001  HF vs HF+Dapa: p<0.001 |
| Serum IL-6 |  |  | Con:  4.36±1.07  HF: 27.17±2.54  HF+Dapa:  6.73±1.55 | Con vs HF: p<0.001  HF vs HF+Dapa: p<0.001 |
| Serum IL-17 |  |  | Con:  0  HF:  51.11±3.12  HF+Dapa:  0 | Con vs HF: p<0.001  HF vs HF+Dapa: p<0.001 |
| LVEDV |  |  | Con at Week 8:  72.29±1.76  HF at Week 8  96.33±3.29  HF+Dapa at Week 8:  82.66±2.52 | Con vs HF: p<0.001  HF vs HF+Dapa: p<0.01 |
| LVESV |  |  | Con at Week 8:  37.86±4.87  HF at Week 8  84.26±5.15  HF+Dapa at Week 8:  57.30±5.20 | Con vs HF: p<0.001  HF vs HF+Dapa: p<0.01 |
| LV mass  (mg) |  |  | Con:  136.23±14.06  HF:  183.96±18.59  HF+Dapa:  144.21±15.51 | Con vs HF: p<0.01  HF vs HF+Dapa: p<0.05 |
| LV mass/body mass  (mg/g) |  |  | Con:  6.25±0.43  HF:  7.40±0.38  HF+Dapa:  6.24±0.39 | Con vs HF: p<0.05  HF vs HF+Dapa: p<0.05 |
| FS (%) |  |  | Con at Week 8:  33.31±2.12  HF at Week 8  17.84±2.67  HF+Dapa at Week 8:  27.61±2.51 | Con vs HF: p<0.001  HF vs HF+Dapa: p<0.01 |
| LVEF |  |  | Con at Week 8:  59.21±3.03  HF at Week 8  28.73±3.08  HF+Dapa at Week 8:  45.12±5.42 | Con vs HF: p<0.001  HF vs HF+Dapa: p<0.01 |
| Infraction size  (% of LV) |  |  | Con:  0  HF:  37.11±4.95  HF+Dapa:  22.59±2.27 | Con vs HF: p<0.001  HF vs HF+Dapa: p<0.01 |
| α-SMA |  |  | Con:  0.48±0.07  HF:  0.88±0.07  HF+Dapa:  0.45±0.12 | Con vs HF: p<0.001  HF vs HF+Dapa: p<0.01 |
| FN |  |  | Con:  0.36±0.09  HF:  0.78±0.07  HF+Dapa:  0.51±0.09 | Con vs HF: p<0.001  HF vs HF+Dapa: p<0.05 |
| CTGF |  |  | Con:  0.37±0.05  HF:  0.89±0.08  HF+Dapa:  0.44±0.11 | Con vs HF: p<0.001  HF vs HF+Dapa: p<0.01 |
| Collagen I |  |  | Con:  0.26±0.05  HF:  0.63±0.09  HF+Dapa:  0.25±0.05 | Con vs HF: p<0.01  HF vs HF+Dapa: p<0.01 |
| Collagen III |  |  | Con:  0.24±0.05  HF:  0.67±0.09  HF+Dapa:  0.30±0.06 | Con vs HF: p<0.01  HF vs HF+Dapa: p<0.01 |
| HR |  |  |  |  |
